# Hydrogen-Rich Water Consumption for Acute and Residual Fatigue After Simulated Football Matches: Protocol for a Randomized, Double-Blinded, Placebo-Controlled, Parallel Trial

**DOI:** 10.2196/69744

**Published:** 2025-07-22

**Authors:** Michal Hruby, Karel Hulka, David Bernatik

**Affiliations:** 1 Department of Sport Faculty of Physical Culture Olomouc Palacký University Olomouc Olomouc Czech Republic

**Keywords:** molecular hydrogen, oxidative stress, recovery, repeated sprint ability, antifatigue effect, readiness to play

## Abstract

**Background:**

Football matches induce acute and residual fatigue, impairing neuromuscular, metabolic, and perceptual performance. Hydrogen-rich water (HRW) is a novel intervention with antifatigue and antioxidative properties. The intermittent high-intensity nature of football, which includes frequent accelerations, decelerations, sprints, changes of direction, and physical contacts, imposes substantial demands on both central and peripheral physiological systems. This results in acute fatigue, observable during or immediately after a match, and residual fatigue, which can persist for 24-72 hours post match, depending on the intensity, match context, and recovery strategies.

**Objective:**

This study will investigate the effects of pre-exercise HRW administration versus a placebo on neuromuscular performance, biochemical markers, and perceptual measures of fatigue during a 72-hour recovery after a simulated football match.

**Methods:**

Using a randomized, double-blinded, placebo-controlled, parallel design, elite junior football players will undergo neuromuscular performance assessments (repeated sprint ability and countermovement jump test). Metabolic fatigue will be measured by creatine kinase level and muscle soreness, rated using a visual analog scale. These assessments will occur at critical time points: immediately post warm-up; directly following the simulated football match to detect acute fatigue; and 24, 48, and 72 hours after training sessions to detect residual fatigue.

**Results:**

Data collection has been scheduled with the clubs to coincide with the beginning of the players’ transition period (ie, at the start of August 2025). The expected duration of data collection, including the initial medical examination, is planned to be 1 month. We anticipate publishing the results in late 2025 or during the first half of 2026.

**Conclusions:**

This study will assess the influence of molecular hydrogen on acute fatigue manifestation and recovery quality during a 72-hour period after a simulated football match. The potential positive effects of molecular hydrogen, such as attenuation of oxidative stress, reduction in muscle damage markers, and accelerated neuromuscular recovery, may contribute to faster restoration of functional capacities. If confirmed, these effects could enhance players’ readiness to return to high-intensity training and optimize the structure of microcycles in competitive periods. Additionally, understanding the recovery dynamics facilitated by HRW may inform evidence-based recovery strategies and support individualized player monitoring frameworks. The possible positive effect of molecular hydrogen would speed up the players’ readiness to train after the match and help protect players against illness and noncontact injuries.

## Introduction

Football is one of the most popular sports worldwide, characterized by its dynamic and intermittent nature that involves a combination of high-speed and high-intensity effort interspersed with periods of low-intensity activity [1]. Understanding the physical and physiological demands of football matches is essential for developing effective training strategies [2]. Given the combined pressures of matches, training sessions, and associated stressors, players must sustain optimal psychological and physical readiness, as well as overall health, throughout the season and tournaments to achieve peak performance. Monitoring players’ readiness and residual fatigue post match is therefore critical for practitioners, as it enables the design of tailored recovery and training plans. This comprehensive approach ensures players maintain consistent performance levels while effectively managing the physical and mental challenges of the sport [3].

Football matches generate significant acute fatigue in players, impacting metabolic, neuromuscular, and perceptual parameters [4]. Recognizing these effects is vital for optimizing recovery strategies and enhancing subsequent performance [5]. The physical demands of modern matches have increased, with players experiencing short recovery periods between games and high neuromuscular strain. This includes a greater frequency of high-intensity running and accelerative actions, which can lead to prolonged residual fatigue [6].

Phosphocreatine availability plays a critical role in intermittent and multiple-sprint performance, with its depletion contributing to performance decline during matches [7]. Prolonged intermittent activity leads to adenosine triphosphate (ATP) and phosphocreatine depletion, reduced neuromuscular excitability, and disruptions in ion homeostasis, which increases reliance on aerobic energy production while inhibiting anaerobic pathways [8,9]. In futsal, for example, 93% of energy production is derived aerobically, with only 5% from anaerobic sources [10]. The relationship between maximal oxygen uptake and repeated sprint ability (RSA) remains ambiguous, with some studies suggesting that RSA performance is more influenced by muscle mitochondrial respiration rates than by maximal oxygen uptake [11].

Mitochondria, through aerobic metabolism, produce reactive oxygen and nitrogen species (ROS/RNS), which serve as both signaling molecules and potential causes of oxidative stress [12]. While moderate ROS/RNS levels are essential for muscle adaptation after training [13], excessive production during repeated sprints can lead to peripheral fatigue and delayed recovery [14]. The body’s antioxidant system works to regulate ROS/RNS levels, but intense exercise can overwhelm this system, demonstrating the delicate balance between energy production and oxidative stress in football [15].

Molecular hydrogen (H_2_) has emerged as a promising intervention for sports performance due to its selective antioxidant properties, targeting ROS/RNS produced during aerobic metabolism [16]. H_2_ also exhibits anti-inflammatory and antiapoptotic effects, along with pH-neutralizing and lactate-reducing capabilities, which can enhance mitochondrial respiration and improve performance in repeated sprint activities [17,18]. Hydrogen-rich water (HRW) has been proposed as a practical hydration strategy during exercise, with evidence suggesting its potential to reduce muscle soreness, perceived exertion, and acute fatigue post exercise [19,20].

Postmatch fatigue significantly affects neuromuscular performance, with assessments like the countermovement jump (CMJ) and RSA tests providing valuable insights [21]. Tools such as the visual analog scale (VAS) are also used to evaluate postexercise muscle soreness, offering reliable measures of discomfort linked to tissue damage [22,23].

Creatine kinase (CK) is a reliable indicator of muscle membrane permeability, as it is predominantly located in skeletal and cardiac muscles [24]. Serving as a biochemical marker of muscle damage, CK is found in both the cytosol and mitochondria of muscle cells. Its levels reflect the extent of muscle recovery and readiness for performance, providing valuable insights into a player’s ability to perform after intense physical exertion [25].

The intervals between matches and training sessions may be insufficient for the restoration and recovery of critical processes such as glycogen replenishment, muscle repair, and mental fatigue. This insufficiency not only decreases the likelihood of achieving optimal performance but also elevates the risk of injury [26]. The timeframe required for a player to regain readiness for training following a match is, therefore, a critical factor in planning microcycles. Accordingly, this study aims to evaluate the impact of a simulated football match on the readiness of young elite football players over a 72-hour recovery period.

## Methods

### Study Aim

Recognizing the critical role of recovery during intermittent or RSA performance, the anti-fatigue effects of H_2_ and its impact on mitochondrial ATP synthesis support the hypothesis that acute pre-exercise HRW administration, compared with a placebo, may positively affect biochemical, perceptual, and neuromuscular performance variables during a 72-hour period after a simulated football match. This study aims to assess the impact of pre-exercise HRW administration compared with a placebo on biochemical, perceptual, and performance variables within 72 hours after a simulated football match. The specific aims are to assess the impact of pre-exercise HRW administration on acute neuromuscular performance decrement, the impact of pre-exercise HRW administration on acute muscle soreness and the CK levels of players, and the impact of pre-exercise HRW administration on residual muscle fatigue and duration of return to readiness to play.

### Study Design

**Figure 1:**
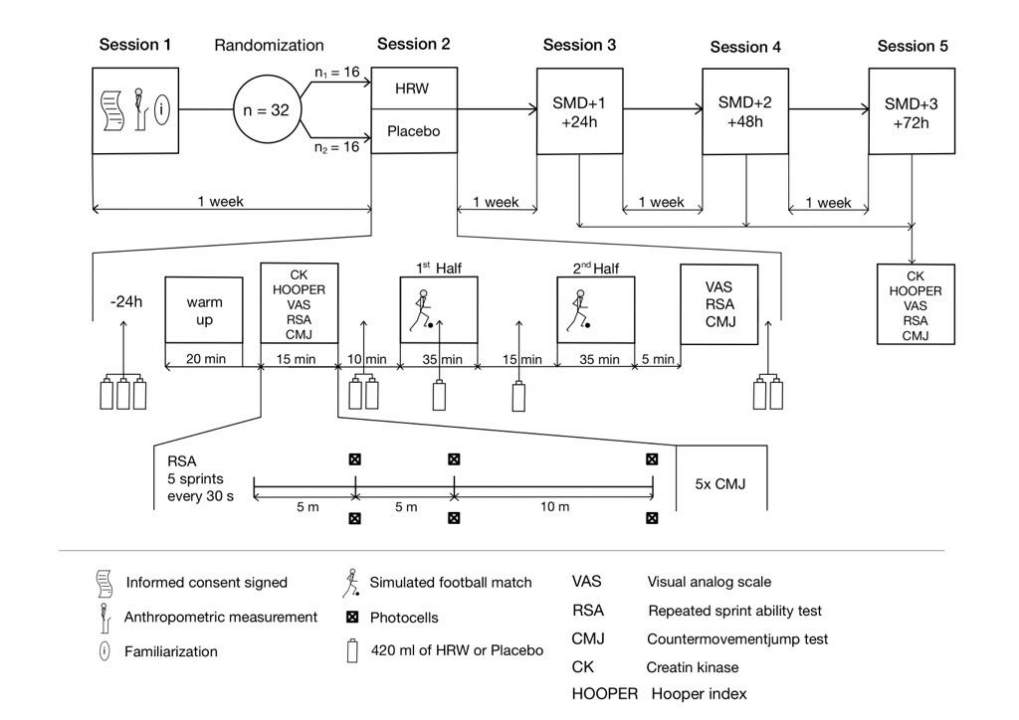
Overview of the study protocol and labeling of sessions. HRW: hydrogen-rich water; SMD: simulated match day.

This study protocol was designed according to the 2023 SPIRIT (Standard Protocol Items: Recommendations for Interventional Trials) statement [27]. The experimental study will use a robust research design, adopting a randomized, placebo-controlled, double-blinded, parallel structure (see Figure 1 and Multimedia Appendix 1).

To ensure unbiased results, the administration of HRW and the placebo was randomized and counterbalanced. The investigation will be conducted over a carefully planned 2-week period on a grass football field. During the initial session, participants will be familiarized with the Team^2^Pro Polar suite (1.4.5 version) and briefed on the regulations governing a simulated football match. Before the first measured session, participants will be stratified based on their playing positions (defenders, midfielders, and forwards) and subsequently randomized into either the HRW or placebo groups. Neuromuscular performance will be assessed through measures including the 20-meter repeated sprint test and CMJ. Metabolic fatigue will be measured through CK levels and muscle soreness, as reported using a VAS. These assessments will occur at critical time points: during simulated match day (SMD) immediately post warm-up; directly following the simulated football match to detect acute fatigue; and immediately post warm-up at intervals of 24 (SMD + 1 day), 48 (SMD + 2 days), and 72 (SMD + 3 days) hours after training sessions to detect residual fatigue. The first measurement sessions will be systematically scheduled on Monday afternoons, allowing for a standardized 72-hour recovery period following the last training session. To enhance ecological validity, the simulated match was designed according to official football rules officiated by an experienced referee, comprising two 35-minute halves, with 11 players per team and no substitutions allowed. Regarding intensity regulation, all participants will be instructed to perform at match intensity, which will be monitored using heart rate (HR) data and rate of perceived exertion scores. These measures will indicate consistent physiological workload across participants, supporting the comparability of internal load responses. Environmental conditions (eg, temperature, lighting) will be held constant throughout all testing sessions to reduce external variability.

### Ethical Considerations

Participants will be informed about the aims of the study, including any risks, discomforts, and benefits, and provide written informed consent. The study was approved by the institutional ethics committee, which follows the Declaration of Helsinki. Participants were not compensated.

### Participants

#### Inclusion and Exclusion Criteria

Czech elite junior male football players will be included. Participants who have at least 10 years of experience, who train at least five times a week with their teams, and who have a match every weekend will be included. Only participants free from injury and medical difficulties will be included in the study. Participants with an illness or injury in the past 3 weeks before the start of measurements and participants who explicitly declined to participate will be excluded.

#### Randomization

A stratified block randomization approach will be used to allocate participants into intervention groups. Participants will be stratified based on their playing position into three distinct strata: (1) defenders, (2) midfielders, and (3) forwards. Within each stratum, participants will be randomly assigned in a 1:1 ratio to either the HRW group or the placebo group using a fixed block randomization method. Randomization sequences will be generated using a robust randomization algorithm, as described by Tu and Benn [28], and implemented via a secure web-based allocation system.

Following participant selection and baseline assessment, each participant will receive either HRW or a placebo in visually identical plastic-aluminum packs, with group allocation determined by the pregenerated randomization code. This process ensures allocation concealment and maintains blinding throughout the study.

#### Blinding

A double-blind design will be implemented to minimize potential bias. The type of water (HRW or placebo) will be packaged identically, with only distinct batch numbers differentiating them. These batch numbers will be coded and maintained solely by the manufacturer, remaining undisclosed until after data collection and statistical analysis. Due to the physical properties of H_2_—being colorless, odorless, and tasteless—participants will be unable to distinguish between the HRW and placebo by sensory perception. Both the players and their coaches, as well as the researchers responsible for data collection and outcome assessment, will be blinded to group allocation throughout the study. This procedure ensures allocation concealment and maintains the integrity of the blinding across all phases of the trial.

#### Sample Size and Power Analysis

The sensitivity analysis was performed using G*Power (Version 3.1, Heinrich-Heine-Universität). Considering a type 1 error of 0.05 (α=.05) and a test power of 80% (β=.15), the calculated sample size for repeated measures (within and between interaction) is 22 participants with 11 in each group. Considering a 10% sample loss, a total of 24 participants will be recruited for the study.

### Data Collection

#### Preparation of HRW and Placebo

HRW was obtained in 420 mL plastic-aluminum packs with a gas-tight cap (Aquastamina-R, Nutristamina, Ostrava, Czech Republic). Chemical characteristics of the HRW and placebo were determined using the pH/Oxidation-Reduction Potential/temperature meter (AD14, Adwa Instruments, Szeged, Hungary). The concentration of the dissolved H_2_ was determined using the H_2_Blue reagent (H_2_ Sciences, Henderson, Nevada) using the manufacturer’s guidelines. HRW/placebo characteristics were as follows: pH=7.9/7.7; oxidation-reduction potential −652/+170 mV; and dissolved H_2_ concentration 1.2-1.4/0.0 ppm. The placebo was obtained from the HRW manufacturer and was produced similarly to Aquastamina-R and packed in the same packs.

The participants were instructed to consume three plastic-aluminum packs (1260 mL) 24 hours before the simulated match, two plastic-aluminum packs (840 mL) after finishing the prematch measurement, one plastic-aluminum pack (420 mL) during the first half, and two plastic-aluminum packs (840 mL) consumed after the postmatch measurement. A total of 3780 mL of water was saturated.

#### RSA Measurement

Participants will complete five 20 m sprints (approximately 3 seconds) departing every 20 seconds with an active recovery (walk back to start). Two seconds before starting each sprint, the participants will be asked to assume the start position. The electronic timing gates (Brower Timing Systems, Draper, Utah) will be set at the start line, after 5 m and 10 m, and at the finish line at 20 m, and always 0.70 m above the surface. According to Pyne et al’s [29] recommendations, 3 scores will be calculated for the total time as the sum of the 5 sprints.

#### CMJ Test and VAS Scale

The CMJ test will be performed on a hardwood indoor surface, commonly used in sports science settings to ensure consistency and reliability of performance measurements. Participants performed 5 jumps and will be instructed to maximize jump height and minimize ground contact time with indoor football shoes. Participants start from an upright standing position with their hands on their hips. The CMJ test will be performed on a mobile optical device, the OptoJump system (Microgate SRL, Italy). Participants will perform three repetitions with a 3-minute recovery; the best attempt will be used. The average height and reactive strength index will be calculated. Reactive strength index will be calculated as jump time divided by contact time [30].

The self-reported perceptual measure by the VAS will be applied to assess lower limb muscle pain [31]. The VAS will be applied just after the CMJ test on a horizontal 100 mm length line with the left and right ends of the line corresponding to “no soreness during repeated maximal jumps” and “most soreness ever experienced during repeated maximal jumps.”

#### HR and Activity Demands

HR will be continuously recorded in 1-second intervals during the simulated match and all training sessions using a Polar Team^2^Pro System (Polar Electro, Kempele, Finland), whereby participants will have HR monitors affixed to their chest and level with the xiphoid process. HR responses will be presented relative to each participant’s individualized maximum HR from laboratory measurements. Raw data from the HR monitors will be exported into Excel (v16.35; Microsoft Corporation). To express training impulse, Edwards summated HR zones (SHRZ) will be calculated as 
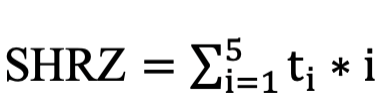
, where *t_i_* is time spent in 5 HR zones (zone 1: 50%-60% of HR_max_; zone 2: 60%-70% of HR_max_; zone 3: 70%-80% of HR_max_; zone 4: 80%-90% of HR_max_; zone 5: 90%-100% of HR_max_). The external load will be calculated as the total distance covered, high-intensity distance [32] as distance covered at a speed >14.4 km·h^−1^, and PlayerLoad as 
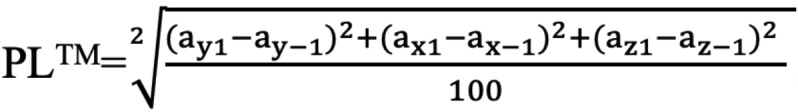
, where *a_y_* is the straight acceleration vector, *a_x_* is the side acceleration vector, and *a_z_* is the vertical acceleration vector.

#### Statistical Analyses

Statistical analysis and data management will be undertaken using SPSS version 22.0 software (IBM Corp), using an α≤.05. The normality and homogeneity of all data will be verified with the Shapiro-Wilk and Levene tests. Mean and SD will be calculated for each outcome measure with a parametric distribution and medians and quartiles for data with a nonparametric distribution. A repeated-measures ANOVA with one random factor as subject, two fixed factors as type of water (HRW/placebo) and time (prematch; postmatch; and 24, 36, and 72 hours post match), and mutual interaction of water and time will be used to assess acute and residual fatigue. When a significant effect of time or water is observed, pairwise comparisons will be performed using Tukey post hoc test. η^2^_p_ was used as a measure of effect size for each analysis of variance, and values were interpreted as having a small effect (0.01≤η^2^_p_<0.06), medium effect (0.06≤η^2^_p_<0.14), and large effect (η^2^_p_≥0.14).

## Results

Data collection has been scheduled with the clubs to coincide with the beginning of the players’ transition period (ie, the start of August 2025). The expected duration of data collection, including the initial medical examination, is planned to be 1 month. We anticipate publishing the results in late 2025 or during the first half of 2026.

## Discussion

### Anticipated Findings

The recovery period between matches and training sessions may not adequately support critical physiological and psychological recovery processes, such as glycogen repletion, muscle damage, and mental fatigue [33]. Insufficient recovery can decrease performance and elevate injury risk. Understanding the time required for players to regain optimal training readiness is essential for designing effective microcycle plans. For this reason, the study will aim to assess the impact of pre-exercise HRW administration compared with a placebo on biochemical, perceptual, and performance variables after a simulated football match within 72 hours.

RSA performance has been shown to lead to a reduction in oxidative stress and may increase antioxidant protection as a specific training adaptation [34]. Research has shown that increased levels of oxidative stress can result in decreased muscle performance after repeated eccentric exercise [35] as a muscle fatigue manifestation. According to Çakir-Atabek et al [35], HRW consumption may enhance the endogenous antioxidant capacity to respond to the intensity-dependent mitochondrial production of ROS, reduce oxidative stress, and enhance mitochondrial ATP production [17], due to the decrease in lactate accumulation [36]. Botek et al [37] showed that H_2_ application induced an increase in mitochondrial membrane potential, increasing oxygen consumption; cellular ATP, which is an indicator of stimulated oxidative phosphorylation; and mitochondrial ATP production. To protect the quality of the H_2_ against the negative effects of increased ROS/RNS, HRW could increase the mitochondrial efﬁciency and peak muscle power output in later stages of the repeated sprints and delay acute muscle fatigue.

### Limitations

First, the simulated football match was designed to replicate competitive conditions, and it may not fully capture the psychological and physiological demands of actual competition. The absence of real opponent-based risks could influence the intensity, motivation, and stress levels of participants, potentially affecting the generalizability of the findings to real match scenarios. Second, participants will be restricted to Czech elite junior male football players. Third, Calbet et al [38] showed a sexual dimorphism in mitochondrial respiratory control, with women displaying larger mitochondrial volume density and higher capacity to oxidize fat, and women contribute to an enlarged O_2_ extraction capacity compared to men. It is important that our results relate to men only. Fourth, blood lactate concentrations will not be analyzed. Fifth, while assessing fatigue over 72 hours is valuable, the recovery period may not capture longer-term effects of HRW on performance and biochemical markers.

## Appendix

Multimedia Appendix 1 Schedule of enrollment as recommended by the 2013 SPIRIT (Standard Protocol Items: Recommendations for Interventional Trials) statement.

## Abbreviations

ATP: adenosine triphosphate
CK: creatine kinase
CMJ: countermovement jump
H2: molecular hydrogen
HR: heart rate
HRW: hydrogen-rich water
ROS/RNS: reactive oxygen and nitrogen species
RSA: repeated sprint ability
SHRZ: summated heart rate zone
SMD: simulated match day
SPIRIT: Standard Protocol Items: Recommendations for Interventional Trials
VAS: visual analog scale

## Data Availability

All research data will be collected, stored, and processed in compliance with ethical standards, data protection regulations, and the Findable, Accessible, Interoperable, and Reusable (FAIR) principles, following the Horizon Europe guidelines. Anonymized datasets will be securely archived in Zenodo, each assigned a permanent DOI and described with standard metadata. A data management plan will be submitted with the initial progress report and updated as needed throughout the project.
