# Supplementary material for: Hydrogen-Rich Water Consumption for Acute and Residual Fatigue After Simulated Football Matches: Protocol for a Randomized, Double-Blinded, Placebo-Controlled, Parallel Trial
Source: JMIR Res Protoc. 2025 Jul 22;14:e69744. doi: 10.2196/69744 (PMC12326158; doi:10.2196/69744)
Supplement: Multimedia Appendix 1 [file resprot_v14i1e69744_app1.docx]

**Table 1.** SPIRIT figure for the schedule of enrolment as recommended by 2013 SPIRIT statement.

| **STUDY PERIOD** | | | | | | | | | | | |
| --- | --- | --- | --- | --- | --- | --- | --- | --- | --- | --- | --- |
|  | **Enrolment** | **Allocation** | **Post-allocation** | | | | | | | | **Close-out** |
| **TIMEPOINT** |  |  | ***2 w before***  ***SM*** | ***24 h before SM*** | ***Before SM*** | ***During SM*** | ***After SM*** | ***24 h after SM*** | ***48 h after SM*** | ***72 h after SM*** | ***July 2025*** |
| **ENROLMENT:** |  |  |  |  |  |  |  |  |  |  |  |
| **Eligibility screen** | X |  |  |  |  |  |  |  |  |  |  |
| **Medical examination** |  |  | X |  |  |  |  |  |  |  | X |
| **Informed consent** | X |  |  |  |  |  |  |  |  |  |  |
| **Antropometric measure** |  |  | X |  |  |  |  |  |  |  | X |
| **Allocation** |  | X |  |  |  |  |  |  |  |  |  |
| **INTERVENTIONS:** |  |  |  |  |  |  |  |  |  |  |  |
| ***Water consumption*** |  |  |  | X | X | X |  |  |  |  |  |
| **ASSESSMENTS:** |  |  |  |  |  |  |  |  |  |  |  |
| ***Peak heart rate testing*** |  |  | X |  |  |  |  |  |  |  |  |
| ***Questionaires, fitness testing*** |  |  |  |  | X |  | X | X | X | X |  |
| ***External and internal load measurement*** |  |  |  |  |  | X |  |  |  |  | X |
| ***Statistical Analysis*** |  |  |  |  |  |  |  |  |  |  | X |
